# Increased phosphatase regenerating liver-1 trigger vascular remodeling in injured ovary via platelet-derived growth factor signaling pathway

**DOI:** 10.1186/s13287-022-02772-9

**Published:** 2022-03-07

**Authors:** Hyeri Park, Jin Seok, Jun Hyeong You, Jae Yeon Kim, Ja-Yun Lim, Gi Jin Kim

**Affiliations:** 1 Department of Biomedical Science, CHA University, 335 Pangyo-Ro, Bundang-Gu, Seongnam-si, Gyeonggi-Do 13488 Republic of Korea; 2 Research Institute of Placenta Science, CHA University, Seongnam-si, Gyeonggi-do 13488 Republic of Korea; 3 Department of Health and Environmental Science, Korea University, 145 Anam-Ro, Seongbuk-Gu, Seoul, 02481 Republic of Korea

**Keywords:** Placenta-derived mesenchymal stem cells, Phosphatase regenerating liver-1, Ovary, Folliculogenesis, Vascular remodeling, Platelet-derived growth factor

## Abstract

**Background:**

Vascular abnormalities in the ovary cause infertility accompanied by ovarian insufficiency due to a microenvironment of barren ovarian tissues. Placenta-derived mesenchymal stem cells (PD-MSCs, Naïve) treatment in ovarian dysfunction shows angiogenic effect, however, the therapeutic mechanism between ovarian function and vascular remodeling still unclear. Therefore, we examined whether by phosphatase regenerating liver-1 (PRL-1), which is correlated with angiogenesis in reproductive systems, overexpressed PD-MSCs could maximize the angiogenic effects in an ovarian tissues injured of rat model with partial ovariectomy and their therapeutic mechanism by enhanced vascular function via PDGF signaling.

**Methods:**

PD-MSCs^PRL-1^ (PRL-1) were generated by nonviral AMAXA gene delivery system and analyzed the vascular remodeling and follicular development in ovary. One week after Sprague–Dawley (SD) rats ovariectomy, Naïve and PRL-1 was transplanted. The animals were sacrificed at 1, 3 and 5 weeks after transplantation and vascular remodeling and follicular development were analyzed. Also, human umbilical vein endothelial cells (HUVECs) and ovarian explantation culture were performed to prove the specific effects and mechanism of PRL-1.

**Results:**

Vascular structures in ovarian tissues (e.g., number of vessels, thickness and lumen area) showed changes in the Naïve and PRL-1-overexpressed PD-MSC (PRL-1) transplantation (Tx) groups compared to the nontransplantation (NTx) group. Especially, PRL-1 induce to increase the expression of platelet-derived growth factor (PDGF), which plays a role in vascular remodeling as well as follicular development, compared to the NTx. Also, the expression of genes related to pericyte and vascular permeability in arteries was significantly enhanced in the PRL-1 compared to the NTx (*p* < 0.05). PRL-1 enhanced the vascular formation and permeability of human umbilical vein endothelial cells (HUVECs) via activated the PDGF signaling pathway.

**Conclusions:**

Our results show that PRL-1 restored ovarian function by enhanced vascular function via PDGF signaling pathway. These findings offer new insight into the effects of functionally enhanced stem cell therapy for reproductive systems and should provide new avenues to develop more efficient therapies in degenerative medicine.

**Supplementary Information:**

The online version contains supplementary material available at 10.1186/s13287-022-02772-9.

## Introduction

Vascular development plays a crucial role in follicle maturation and oxygen, nutrient and hormone substrate delivery  [1, 2]. During maturation, the vascular sheath develops along with the maturation of follicles from primordial to secondary follicles and consists of two concentric networks of vessels in the theca interna and externa layers. Arteries and venules branch into the single-layer capillary plexus of the theca interna, but they are not traversed by capillaries into the basement membrane or the granulosa layer. Moreover, the granulosa layer and other cells forming follicles in the ovary receive hormones and nutrients by diffusion from the peripheral theca layer [3]. In particular, vascular endothelial growth factor (VEGF) and platelet-derived growth factor (PDGF) are major factors involved in follicular development as well as angiogenic factors. Both VEGF and PDGF play a role in stimulating growth to induce the transition of primordial to secondary follicles [4, 5]. During the menstrual cycle, vascular function degenerates, similar to other organs, and as women age, the major function is lost. Moreover, early vascular dysfunction is considered a factor in women with premature ovarian failure (POF) and ovarian dysfunction. Deficiency of the vasculature in the ovary plays a role in regression and causes ovarian dysfunction by decreasing the diffusion of nutrients and hormones into the follicles. Infertility with side effects (e.g., osteoporosis) occurs due to imbalanced hormone levels through limitation of follicle growth for maturation and acceleration of follicular atresia (i.e., apoptotic follicles) [6]. For the treatment of ovarian dysfunction by aging, hormone replacement therapy (HRT) is mainly used, but this treatment shows temporal effects on ovarian reserve as well as increased risk of various conditions such as breast cancer, cardiovascular disease and osteoporosis [7]. Additionally, whether HRT restores ovarian function through vascular remodeling has not been reported.

To overcome the limitation of HRT due to increased disease risk, many scientists have studied whether the paracrine effects of stem cells, including those mediated by several cytokines [e.g., PDGF, VEGF and fibroblast growth factors (FGFs)] secreted by stem cells, could recover ovarian function in animal models of POF [8, 9]. Additionally, therapy using stem cells was reported to restore ovarian function, including improvements in follicular development, regulation of reproductive hormones and attenuation of apoptosis [10]. In previous reports, Cho and their colleagues demonstrated that placenta-derived mesenchymal stem cells (PD-MSCs) secreted various growth factors including VEGF and PDGF, and secreted VEGF from PD-MSCs regulates regeneration of vascular remodeling and stimulates follicular development in rats with ovarian dysfunction [11].

Protein tyrosine phosphatases (PTPs) are dual specificity modulators that regulate cell differentiation and proliferation due to their oncogenic effects. In particular, phosphatase of regenerating liver-1 (PRL-1), called PTP4A1, was reported to be involved in liver regeneration [12]. Recently, Schmidt et al. reported that the role of PRL-1 in ovarian function and reported its responses to follicle-stimulating hormone (FSH), which is critical for steroid production and oocyte maturation in the ovary, in primary Sertoli cell cultures [13]. Moreover, PRL deletion limited angiogenesis (e.g., developmental angiogenesis and sprouting angiogenesis) by inhibiting endothelial cell migration and VEGF signaling [14, 15]. However, the effect of PRL-1 on vascular remodeling in rats with ovarian dysfunction remains unclear.

Therefore, the objective of this study was to demonstrate the therapeutic mechanism by which PRL-1-enhanced PD-MSCs affected vascular remodeling as well as ovarian function and whether the paracrine effects of PDGF secretion could improve ovarian function in ovariectomized (OVX) rats.

## Materials and methods

### Cell culture and gene transfection

Human placental samples for research purposes were approved by the Institutional Review Board of CHA Gangnam Medical Center, Seoul, Republic of Korea (IACUC-190048). Briefly, PD-MSCs were isolated from the chorionic plate of the placenta as described in previous reports [16]. PD-MSCs and PD-MSCs^PRL-1^ were cultured in alpha-minimum essential medium (α-MEM; HyClone, Utah, USA) containing 10% fetal bovine serum (FBS; Gibco-BRL, Oklahoma, USA), 1% penicillin/streptomycin (P/S; Gibco-BRL), 25 ng/ml FGF-4 (Peprotech, New Jersey, USA), and 1 μg/ml heparin (Sigma-Aldrich, Missouri, USA) at 37 °C in a humidified atmosphere of 5% CO_2_. For overexpression of human PRL-1 in Naïve PD-MSCs, a human PRL-1 plasmid containing the CMV6-AC vector (Origene, Inc., Rockville, MD, USA) was transfected into Naïve PD-MSCs using the 4D AMAXA Nucleofector™ system (Lonza, Basel, Switzerland) according to previous reports [17]. After harvest, PD-MSCs were labeled with a PKH67 Fluorescent Cell Linker Kit (Sigma-Aldrich). PD-MSCs (i.e., Naïve) and PD-MSCs^PRL-1^ (i.e., PRL-1) at doses of 5 × 10^5^ cells were injected intravenously via the tail vein in the transplanted animal group.

### Construction of the OVX rat model and transplantation of stem cells

Female 7-week-old Sprague–Dawley rats (Orient Bio, Inc., Seongnam-si, Republic of Korea) were maintained in an air-conditioned animal facility. The rats were housed in groups of two rats per plastic cage with corn-cob bedding and were provided ad libitum access to standard commercial food and tap water. The temperature was 21 °C, and a 12 h/12 h light–dark cycle was employed. The experimental procedures for the animal modeling and experiments were approved by the Institutional Animal Care and Use Committee of CHA University, Seongnam-si, Republic of Korea (IACUC-190007). The ovariectomy was performed by surgically removing one of the ovaries under general anesthesia with avertin (2,2,2-tribromoethanol, Sigma-Aldrich). Each rat was anesthetized through abdominal injection and avertin before the operation. Each group, including the normal, NTx and Tx groups, consisted of 5 rats. One week after OVX modeling, Naïve and PRL-1 (5 × 10^5^ cells, 10–13 passages) labeled with a PKH67-linked kit (Sigma-Aldrich) were injected intravenously through the tail vein. All rats were sacrificed after 1, 3, and 5 weeks to harvest their ovarian tissues and blood samples.

### RNA isolation and quantitative real-time polymerase chain reaction

Total RNA isolated from rat ovarian tissues using TRIzol reagent (Ambion, Thermo Fisher Scientific) according to the manufacturing’s protocol. Total RNA concentration was measured using a Nanodrop spectrophotometer (Thermo Fisher Scientific, Waltham, USA). Total RNA was reverse transcribed into cDNA using Superscript III reverse transcriptase (Invitrogen). The PCR conditions for the synthesis of cDNA were as follows: 5 min at 65 °C, 1 min at 4 °C, 60 min at 50 °C, and 15 min at 72 °C. The cDNA was used for qRT-PCR analysis. It was performed with SYBR Ex Taq (Roche, Basel, Switzerland). The cDNA was subsequently amplified by PCR under the following conditions: 5 s at 95 °C and 40 cycles of 95 °C for 5 s and 60 °C for 30 s. The sequences of the qRT-PCR primers are listed in Additional file 1: Table S1. rGAPDH was used as an internal control for normalization, and each sample was analyzed in triplicate.

### Ovarian explant ex vivo culture

For ovarian tissue cultivation, Matrigel (Corning, New York, USA) was added to a 24-well culture plate for 3 h. Then, each ovary of 7-week-old female rats was cut and rinsed with saline and DPBS containing 1% penicillin. For analysis of PRL-1 gene function in ovarian tissues, PD-MSCs treated with siRNA-PRL-1 at 50 nM (1 × 10^5^ cells per ovary; Invitrogen) were directly injected into whole ovarian tissues after ovarian cultivation. After 48 h, the supernatant and ovarian tissues were collected for analysis. Next, to analyze the paracrine effect of PRL-1, an indirect cocultivation system was established using an insert system (Falcon). Naïve or PRL-1 cells were cocultured with the di-sectioned ovaries on an 8 μm pore size insert (SPL) and seeded at a density of 1 × 10^4^ cells per insert with medium. For inhibition of PDGF, imatinib mesylate (1 μM; Sigma-Aldrich) was used to inhibit the expression of PDGF, and PDGF recombinant (10 ng/ml; PeproTech) was used to induce the expression of PDGF on the insert with medium. After 48 h, the supernatant and di-sectioned ovaries were collected, and the samples were analyzed.

### Nuclear fraction

Rat ovarian tissues from each group were measured at 20 mg in a 1.5 ml tube and diluted in 100 μl of CERI solution (NE-PER™ Nuclear and Cytoplasmic Extraction Reagents, Thermo Fisher Scientific) with phosphatase inhibitor. Tissues were vortexed and incubated on ice at 10 min. Five microliters of CERII solution (NE-PER™ Nuclear and Cytoplasmic Extraction Reagents, Thermo Fisher Scientific) was diluted in tubes. After vortexing, the samples were placed on ice for 1 min and centrifuged (~ 16,000 g) for 5 min. After centrifugation, the supernatant (cytoplasmic) was transferred to clean prechilled tubes. Pellets were suspended in 50 μl of NER solution (NE-PER™ Nuclear and Cytoplasmic Extraction Reagents, Thermo Fisher Scientific). The samples were on ice and vortexed for 15 s every 10 min for a total of 40 min. Then, the samples were centrifuged at maximum speed (~ 16,000 g) in a microcentrifuge for 10 min. The supernatant (nuclear extract) fraction was transferred to clean prechilled tubes.

### Protein isolation and western blotting

Rat ovarian tissues from each group were homogenized and lysed on ice with RIPA buffer (Sigma-Aldrich) containing protease inhibitor cocktail (Roche) and phosphatase inhibitor cocktail (genDEPOT, Texas, USA). Equal concentrations of protein extracts were separated using 8% sodium dodecyl sulfate–polyacrylamide gel electrophoresis (SDS-PAGE). The separated proteins were transferred onto polyvinylidene difluoride (PVDF) membranes (Bio-Rad Laboratories, California, USA) using a Transfer Turbo system (Bio-Rad Laboratories). Membranes were blocked in blocking buffer (5% BSA) at room temperature for 1 h. Next, the membrane was incubated with primary antibody (1:1000) in 2% BSA at 4 °C overnight. The following antibodies were mixed with 2% BSA and incubated at 4 °C: rabbit anti-PDGF receptor β (28E1; 3169S, Cell Signaling Technology, Danvers, MA, USA) diluted 1:1000, mouse anti-PDGFR-α (C-9; sc-398206, Santa Cruz Biotechnology, Dallas, Texas, USA) diluted 1:100, mouse anti-HIF-1α (28b; sc-13515, Santa Cruz Biotechnology) diluted 1:5000, rabbit anti-CD105 (endoglin; bs-4609R; Bioss Antibodies, Woburn, MA, USA) diluted 1:1000, rabbit-anti VEGF receptor 2 (D5B1; 9698S, Cell Signaling Technology) diluted 1:1000, rabbit anti-total mTOR (7C10; 2983S, Cell Signaling Technology) diluted 1:1000, rabbit anti-mTOR (phospho S2448; ab109268, Abcam; Cambridge; MA; USA) diluted 1:1000, rabbit anti-LC3B (2775S, Cell Signaling Technology) diluted 1:1000, mouse anti-Erg-1/2/3 (D-3; sc-271048, Santa Cruz Biotechnology) diluted 1:1000, mouse anti-Nobox (D-3; sc-390016, Santa Cruz Biotechnology) diluted 1:1000, rabbit anti-BMP15 (MBS2516631, Mybiosource, San Diego, CA, USA) diluted 1:1000, and rabbit anti-EGF receptor (2232S, Cell Signaling Technology) diluted 1:1000. After incubation, the membranes were washed with 1X Tris-buffered saline-Tween 20 (TBS-T) and then incubated with secondary antibody (1:10,000) according to the manufacturer’s instructions at room temperature for 1 h. After the membranes were washed, they were treated with a Clarity Western ECL kit (Bio-Rad Laboratories) at room temperature for 5 min. The protein bands were detected by a ChemiDoc XRS+ imaging system (Bio-Rad Laboratories). The bands were analyzed by the ImageJ program (Wayne Rasband, Bethesda, Maryland). The fold change value of intensity is a comparative value of gene expression.

### ELISA

All blood samples were collected from the aortas of rats in the normal, NTx and Tx groups. Individual serum samples were separated from whole blood by using a blood collection tube (Vacutainer; BD Biosciences, San Jose, CA, USA). All blood serum was stored at − 80 °C, and estrogen (Biovision, Milpitas, CA, USA), AMH (Elabscience Biotechnology, MA, USA), FSH (Abnova, Taipei, Taiwan), and testosterone (Mybiosource, San Diego, CA, USA) activity in serum was analyzed by ELISA kits following the manufacturer’s instructions. In brief, an equal volume of sample was added to the specific antibody-coated plates. Next, specific horseradish peroxidase (HRP)-conjugates were added to each well and incubated at 37 °C. After the substrates had been added and incubated in the dark for substrate development, the antibody activity was analyzed by using a microplate reader (BioTek, Winooski, VT, USA).

### H&E staining for follicle counting

Ovarian tissues were fixed with 10% neutral buffered formalin (BBC, Washington, USA), embedded in paraffin, and serially sectioned into 4 μm ovaries. Sectioned ovarian tissues were deparaffinized in a 60 °C dry oven and by xylene and ethanol. Deparaffinized tissues were washed under tap water. The slides were dipped in Harris hematoxylin (Leica Biosystems, Wetzlar, Germany) for 7 min, dipped in 0.1% HCl for 2 s and counterstained with alcoholic eosin Y solution (Sigma-Aldrich). The stained slides were scanned for whole ovaries by 3D HISTECH (The Digital Pathology Company, Budapest, Hungary). The follicles were counted every 100 μm in serially sectioned slides and were defined as the total follicles, including primordial, primary, secondary, and preovulatory follicles, and the antral follicles according to previous reports [18].

### Immunohistochemistry staining

Sectioned ovarian tissues were deparaffinized in a 60 °C dry oven and by xylene and ethanol. Deparaffinized tissues were subjected to antigen retrieval by EDTA (eLbio, Seongnam-si, Republic of Korea) reaction and slowly cooled with water. The ovarian tissues were washed with distilled water (D. W) and treated with peroxide blocking solution containing 3% H_2_O_2_ in methanol for 10 min. Next, the ovarian tissues were washed with D.W and treated with primary antibodies with diluent buffer (Dako) at 4 °C overnight. The rabbit anti-PDGF receptor β antibody (28E1; 3169S, Cell Signaling Technology) was diluted 1:250. After removal of the unbound primary antibody, the tissues in slides were incubated with Dako Real EnVision HRP Rabbit/Mouse secondary antibody (Dako, California, USA) at room temperature for 1 h. The slides were incubated with DAB and counterstained with hematoxylin (Dako). After reaction, slides were rinsed by tap water. Slides were dehydrated by ethanol and xylene. Tissues were analyzed by the 3D HISTECT program (The Digital Pathology Company).

### Immunofluorescence staining

Frozen ovarian section blocks were sectioned at 6 μm thickness and fixed with methanol for 10 min. After air drying, ovarian tissues were washed with 1X phosphate-buffered saline (PBS) at room temperature 3 times for 5 min each. Then, 1x PBS at the tissue edge was removed and placed in a humidified chamber. Tissues were treated with blocking solution (Dako) at room temperature for 1 h and treated with primary antibodies against each target gene at 4 °C overnight. The following antibodies were mixed with antibody diluent buffer (Dako) and used: goat anti-PECAM-1 (CD31; sc-1506, Santa Cruz Biotechnology) diluted 1:250, rabbit anti-LC3B (2775S, Cell Signaling Technology) diluted 1:200, and rabbit anti-phospho-Src (Tyr527, 2105S, Cell Signaling Technology) diluted 1:200. Next, all tissues were incubated at room temperature for 1 h. The tissues were washed with 1X PBS at room temperature 3 times for 5 min and then treated with secondary antibody (1:250) at room temperature for 1 h. The cells were washed with 1X PBS at room temperature for 5 min 3 times. Then, the tissues were mounted with mounting medium with DAPI (Vectashield, Burlingame, CA, USA). The prepared slides were observed by fluorescence microscopy (Zeiss LSM 780, Oberkochen, Germany) at 40× and 63× magnification. All parts of each slide were observed, and representative images were captured.

### HUVEC permeability dextran assay for vascular function

HUVECs were cultured with ECM medium (Science Cell, California, USA) at 37 °C in a 5% CO2 incubator. Matrigel was coated on an insert with a 0.4 μm pore size (Falcon, New York, USA). After 3 h, HUVECs at a dose of 3 × 10^5^ cells were seeded on an insert with a 0.4 μm pore size with medium. After 24 h, cells were treated with 100 μg/ml 5-FU. After treatment for 48 h, the medium was changed, and Naïve and PRL-1-overexpressing cells were seeded in 24-well plates (Falcon). After coculture for 24 h, inserts were transferred to new wells with medium, and dextran (Sigma-Aldrich) was added to the insert. After 20 min, medium on a 24-well plate was loaded in a 96-well assay black plate (Costar, Washington, USA), and the intensity was read with a Tecan assay.

### Statistical analysis

All experiments were conducted in duplicate or triplicate. The results are presented as the mean ± standard error. Student’s *t*-test was used to analyze the groupwise comparisons. GraphPad Prism 5.0 (GraphPad Software, Inc., CA, USA) was used to conduct statistical analysis using one-way ANOVA followed by Tukey’s multiple comparisons test. *p* values less than 0.05 were considered significant.

## Results

### PRL-1-enhanced PD-MSC transplantation increased the expression of PDGF-BB and PDGFRβ and activation of the PDGF signaling pathway

**Fig. 1:**
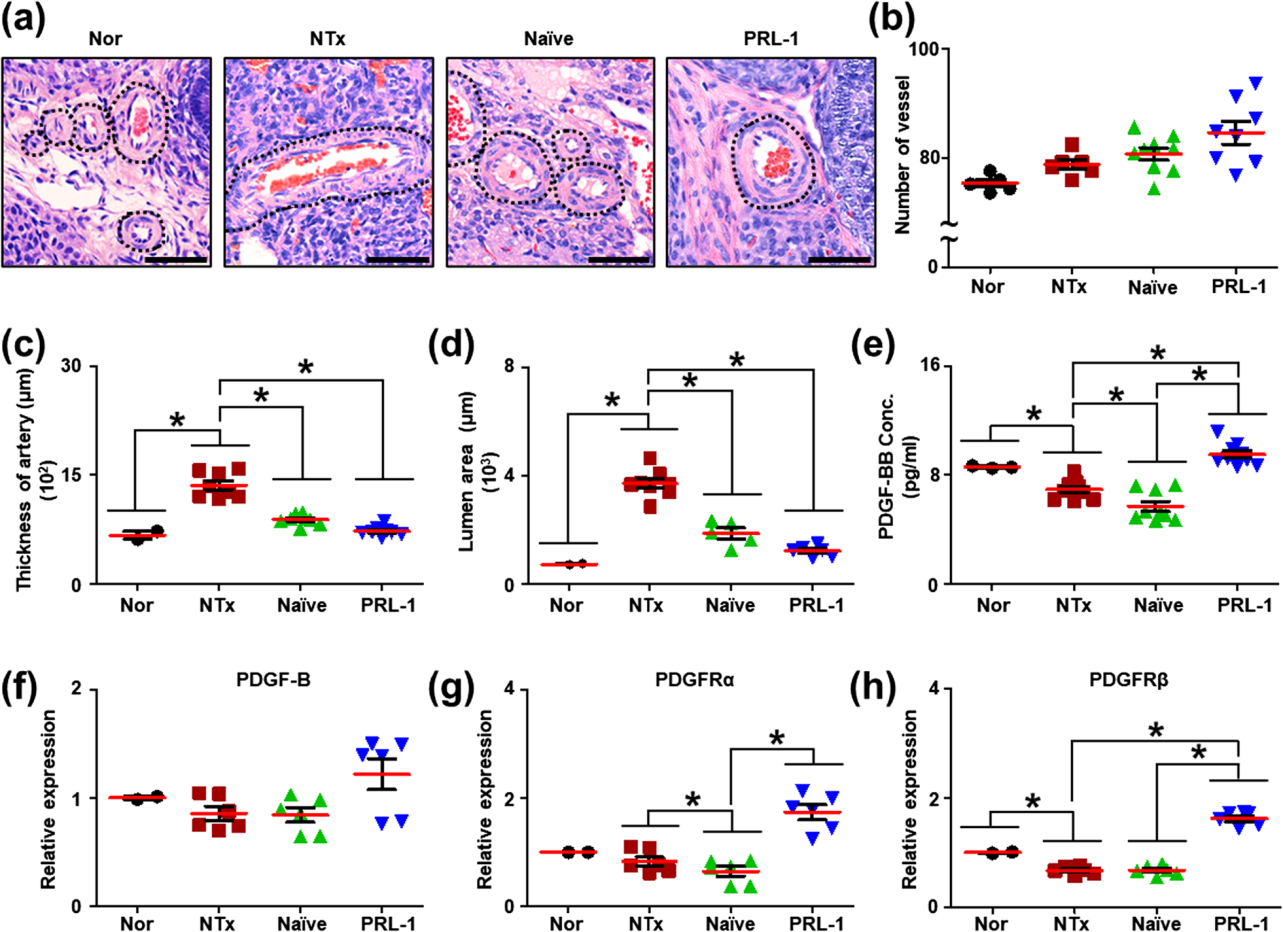
Effect of transplanted PRL-1 on vessels in the ovaries of OVX rats. **a** Histological analysis of the blood vessel structure in ovarian tissues was analyzed by H&E staining. Dotted line: blood vessels. Scale bar: 1.5 mm. Magnification: 20×. **b** The number of vessels, **c** thickness of the artery and **d** area of the lumen were analyzed by the 3D HISTECH program. **e** The concentration of PDGF-BB in serum was analyzed by ELISAs. **f** The mRNA expression of PDGF-B, **g** PDGFRα and **h** PDGFRβ was analyzed by qRT-PCR. The data are representative of three independent experiments and expressed as the mean ± S.D. *p < 0.05, normal versus NTx, NTx versus Tx (Naïve and PRL-1), and Naïve versus PRL-1 at each time point

The development of follicles in ovarian tissues parallels the development of blood vessels, and abnormal development of blood vessels reduces ovarian function [19]. To examine the structure of blood vessels after PRL-1-enhanced PD-MSC transplantation, we analyzed ovarian sections from different groups by hematoxylin and eosin (H&E) staining. In the nontransplantation (NTx) group, we observed that blood vessels were elongated compared to those in the normal group. The restoration of blood vessel structure was observed in the transplantation (Tx) group compared to the NTx group; the blood vessel structure changed from an uneven wall to a regular circular wall (Fig. 1a). To analyze the digitization of the blood vessel structure, we measured the number of vessels, the thickness of the arteries, and the lumen area of the vessels. The number of vessels was increased between the NTx and Tx groups including Naïve and PRL-1 groups compared to the normal group. However, the number of vessels was not significantly different between the groups (Fig. 1b). Interestingly, the thickness of the artery and lumen area were substantially increased in the NTx groups compared to the normal group and significantly reduced in the Tx groups compared to the NTx groups (Fig. 1c, d; *p < 0.05). All Tx groups had vascular structures in the ovaries similar to those of the normal group. These data suggest that transplanted PRL-1-enhanced PD-MSCs changed the structure of blood vessels in the ovaries of the OVX rats.

**Fig. 2:**
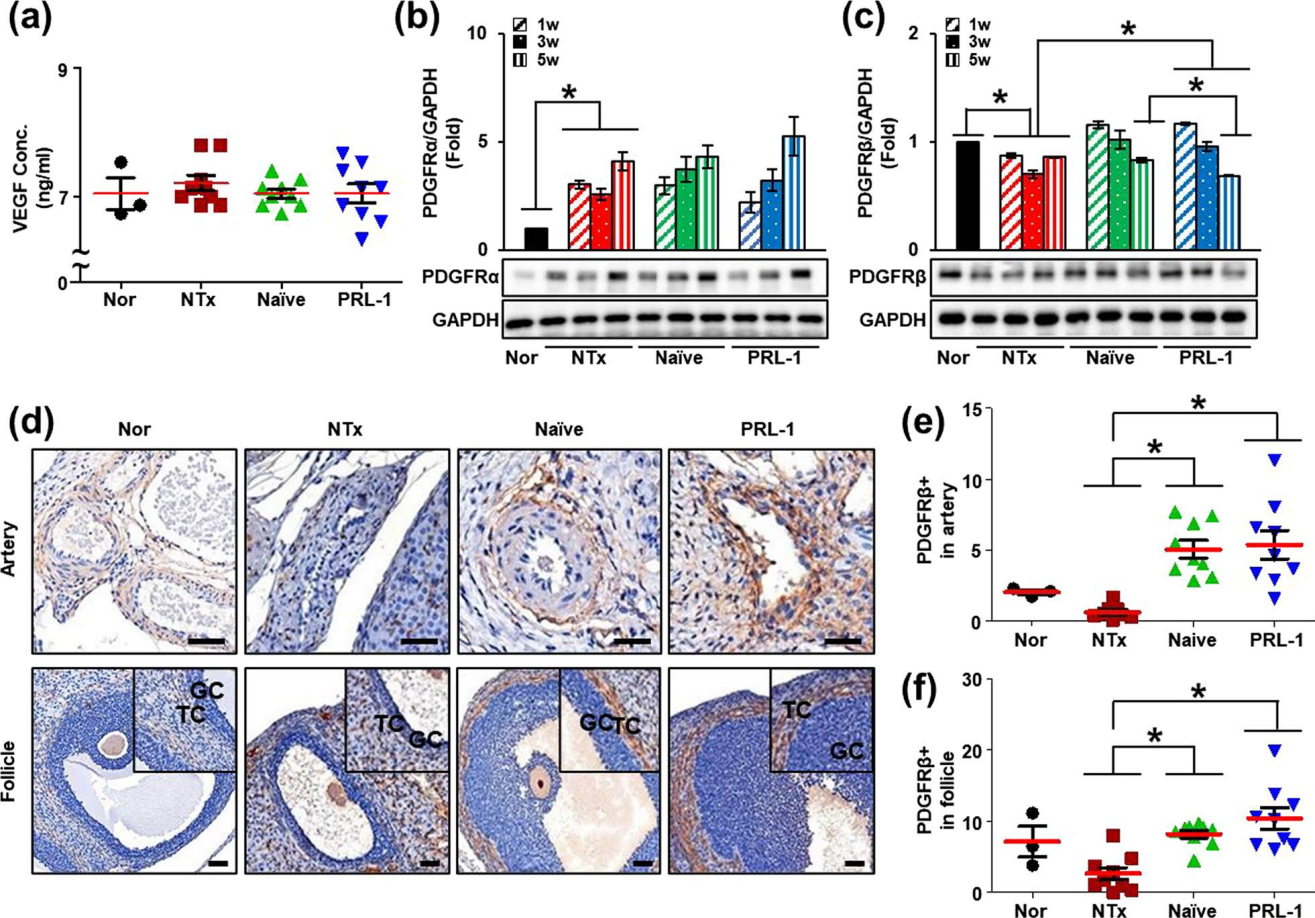
Effect of transplanted PRL-1-overexpressing cells on the PDGF signaling pathway in the ovaries of OVX rats. **a** The concentration of VEGF in serum was analyzed by ELISAs. **b** The protein expression of PDGFRα and **c** PDGFRβ was analyzed by western blots. **d** The localization and gene expression of PDGFRβ in arteries and follicles of ovarian tissues were analyzed by immunohistochemistry staining. **e** The PDGFRβ intensity of arteries and **f** mature follicles in the ovary was analyzed by the 3D HISTECH program. The data are representative of three independent experiments and expressed as the means ± S.D. *p < 0.05, normal versus NTx, NTx versus Tx (Naïve and PRL-1), and Naïve versus PRL-1 at each time point

A previous report suggested that transplanted PD-MSCs improved ovarian function via vascular remodeling through the VEGF pathway [11]. Hence, we analyzed the differences between the Naïve and PRL-1 groups to determine whether changes in vascular remodeling led to improved ovarian function. Basically, it is well known that PDGF is a growth factor that regulates cell growth and division and plays a role in blood vessel formation, angiogenesis and embryonic development. Previously, we examined whether PRL-1-overexpressing PD-MSCs secreted PDGF-BB by using Profiler Human XL Cytokine Arrays. We found that the PDGF-BB level in the supernatant was significantly increased in the Naïve group compared to the PRL-1 group (Additional file 1: Fig. S1a, b). Hence, we investigated the homology of PDGF-BB between humans and rats in the NIH website (http://ncbi.nim.nih.gov), and the similarity was 86%. In the serum of the OVX rats, the level of VEGF, a vascular remodeling factor, showed no difference among the groups (Fig. 2a). For this reason, the level of PDGF-BB, which is a PDGF ligand that plays a role in angiogenesis and pericyte recruitment, was significantly decreased in the NTx and Naïve Tx groups and substantially increased in the PRL-1 Tx groups compared to the Naïve Tx groups (Fig. 1e; *p < 0.05). These data indicated that increased human PDGF-BB by transplanted PRL-1-overexpressing PD-MSCs had an angiogenic effect in the ovary tissues of OVX rats.

Additionally, to analyze the expression of PDGF-B and its receptors (e.g., PDGFRα and PDGFRβ), we assessed the mRNA expression in ovarian tissues. The mRNA expression of PDGF-B was decreased in the NTx and Naïve Tx groups compared to the normal group and increased in the PRL-1 Tx groups compared to the NTx groups (Fig. 1f). Likewise, the mRNA expression of PDGFRα and PDGFRβ was higher in the PRL-1 Tx groups than in the NTx and Naïve groups (Fig. 1g, h; *p < 0.05). Moreover, the protein expression of PDGFRα and PDGFRβ was analyzed in ovarian tissues. The protein expression of PDGFRα was significantly increased in all groups compared to the normal group (Fig. 2b). Interestingly, the protein expression of PDGFRβ was significantly decreased in the NTx groups compared to the normal group and increased in the Naïve and PRL-1 Tx groups compared to the NTx groups at 1 week (Fig. 2c; *p < 0.05). PDGFRα and PDGFRβ are known to regulate follicular development and vascular function, including angiogenesis and blood vessel formation in the ovary.

Hence, we analyzed the PDGF signaling pathway downstream [e.g., protein kinase C-delta (PKCδ), proto-oncogene tyrosine protein kinase Src (Src), VEGF and VEGFR2] to determine whether the PDGF signaling pathway is activated by PRL-1-overexpressing cell transplantation in ovarian tissues of the OVX rats. The mRNA expression of PKCδ, which interacts with PRL-1 and regulates proliferation and migration, was decreased in all groups compared to the normal group (Additional file 1: Fig. S2a). The positive signals of p-Src expression were higher in the PRL-1 Tx groups than in the NTx and Naïve Tx groups at 1 week (Additional file 1: Fig. S2b). Also, we analyzed the expression of the VEGF signaling pathway-induced PDGF gene in rat serum and the mRNA and protein levels in ovarian tissues. The mRNA expression of VEGF and VEGFR2 was significantly decreased in the PRL-1 Tx group compared to the NTx and Naïve Tx groups (Additional file 1: Fig. S2c, d). The protein expression of VEGFR2 was substantially decreased in the NTx groups compared to the normal group and increased in the Naïve and PRL-1 Tx groups compared to the NTx groups at 1 week and 3 weeks (Additional file 1: Fig. S2e). Interestingly, the protein expression of VEGFR2 in the ovary showed a similar pattern to the PDGFRβ gene expression in the ovaries of the OVX rats. Additionally, the mRNA expression of HIF1α and endoglin, which are proangiogenic factors and upstream of the VEGF signaling pathway, was significantly increased in the PRL-1 Tx groups compared to the NTx and Naïve Tx groups (Additional file 1: Fig. S3a, b; *p < 0.05). The protein expression of endoglin in the ovary was similar to PDGFRβ protein expression and increased in the PRL-1 Tx group compared to the NTx and Naïve Tx group at 1 week. However, the protein expression of HIF1α in the ovary was not different (Additional file 1: Fig. S3c, d; *p < 0.05).

However, analysis of angiogenic factors in homogenized whole ovaries is inappropriate for vascular remodeling studies. Hence, we performed immunohistochemical staining to determine whether PDGFRβ was expressed in mature follicles and arteries in ovarian tissues. As shown in Fig. 2d, PDGFRβ was localized in antral follicles and arteries in ovarian tissues. To demonstrate the difference in each group, we analyzed the expression of PDGFRβ in follicles and arteries of ovarian tissues using the 3D HISTECH program. The results showed that PDGFRβ expression in antral follicles and arteries was significantly decreased in the NTx group compared to the normal group and substantially increased in the Naïve and PRL-1 Tx groups (Fig. 2e, f; *p < 0.05). These data indicated that the secreted PDGF from transplanted PRL-1-overexpressing PD-MSCs activates the PDGF signaling pathway in arteries and antral follicles of ovarian tissues.

### PRL-1-overexpressing PD-MSCs transplantation enhanced pericyte recruitment in the arteries of ovariantissues via the PDGF signaling pathway

**Fig. 3:**
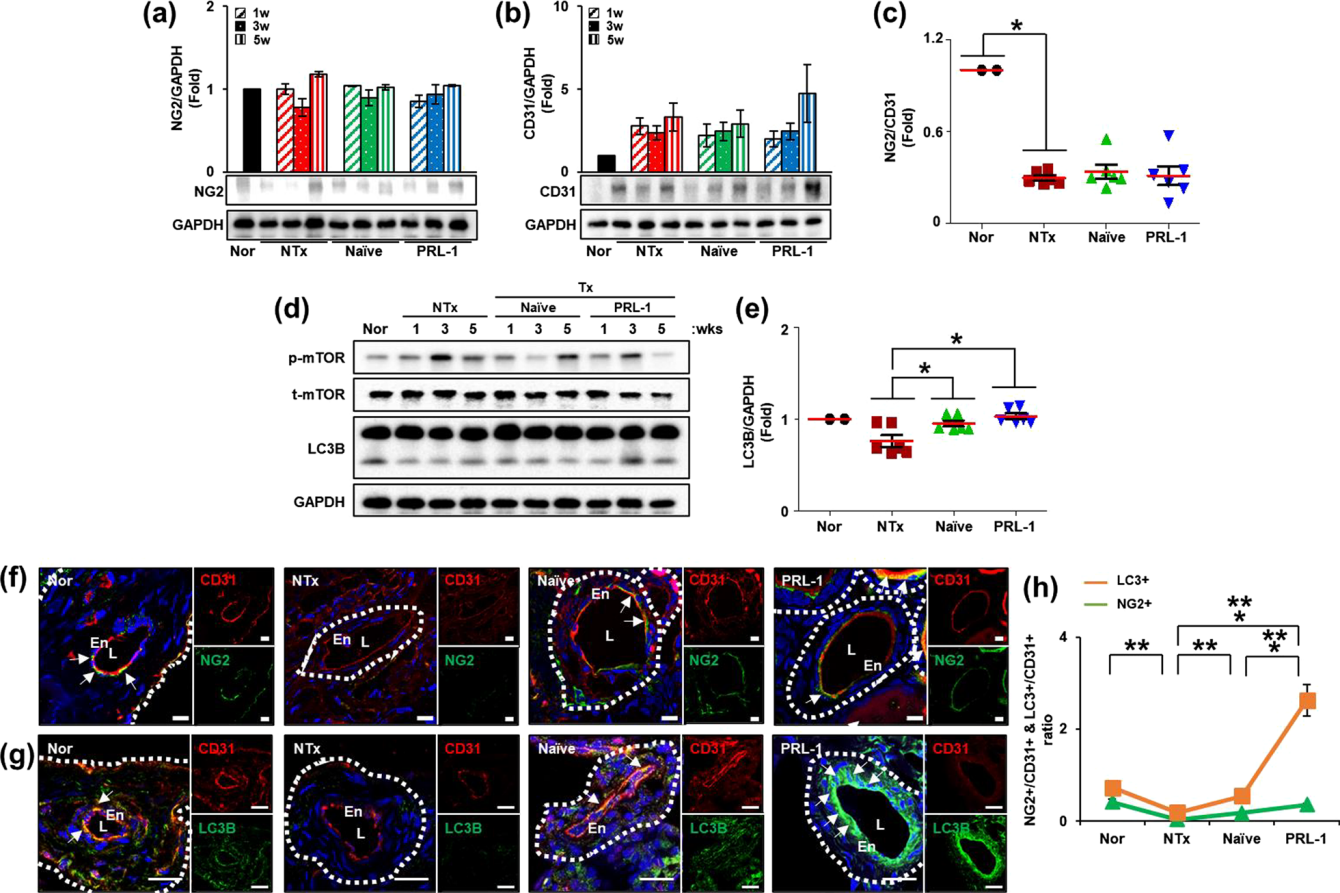
Effect of transplanted PRL-1-overexpressig cells on pericyte recruitment in the ovaries of OVX rats. **a** The protein expression of NG2 and **b** CD31 and **c** the ratio of NG2/CD31 were analyzed by western blots. **d** The gene expression of mTOR and LC3B and **e** the intensity of LC3B gene expression were analyzed by western blots and the ImageJ program. **f** The gene expression and localization of NG2 and CD31 and **g** LC3B and CD31 in arteries of ovarian tissues were analyzed by immunofluorescence staining. **h** The ratio by fluorescence intensity of NG2 and LC3B was analyzed by the ImageJ program. The data are representative of three independent experiments and expressed as the mean ± S.D. *p < 0.05, normal versus NTx, NTx versus Tx (Naïve and PRL-1), and Naïve versus PRL-1 at each time point

Pericyte coverage induced by PDGF, which is the main factor related to pericyte recruitment in blood vessels, is required for the stabilization of immature endothelial tubes [20, 21]. Hence, we confirmed the expression of pericytes and endothelial cells through the ratio of neuron-glial antigen 2 (NG2) and cluster of differentiation 31 [CD31; platelet endothelial cell adhesion molecule (PECAM-1)] in ovarian tissues. As a result, the ratio of NG2 to CD31 in ovarian tissues was decreased in the NTx groups compared to the normal group and increased in the Naïve and PRL-1 Tx groups (Fig. 3a–c). To analyze the ratio of NG2 to CD31, which are representative markers for pericyte, in vessels of ovarian tissues, we performed immunofluorescence staining of ovarian tissues from the OVX rats. As shown in Fig. 3f, the expression of NG2 and CD31 was localized in the pericytes and endothelium of vessels. To quantify positive signals, we analyzed the ratio between NG1 and CD31 expression in vessels using the ImageJ program. The ratio of NG2 to CD31 expression in vessels was significantly decreased in the NTx group compared to the normal group and increased in the PRL-1 group compared to the Naïve group (Fig. 3h; *p < 0.05). These data suggested that transplanted PRL-1-overexpressing PD-MSCs induced pericyte recruitment of vessels for vascular remodeling in the ovaries of the OVX rats.

Next, we analyzed autophagy during vascular regeneration in endothelial cells to demonstrate vessel stability by pericyte recruitment. To analyze vascular remodeling through the autophagic signaling pathway, we analyzed the expression of genes related to the autophagic signaling pathway (e.g., LC3B and mTOR) in the ovarian tissues of the OVX rats (Fig. 3d). The protein expression of LC3B, which is an autophagic regulator, was significantly increased in the ovary in the PRL-1 Tx groups compared to the NTx and Naïve Tx groups (Fig. 3e; *p < 0.05). To demonstrate LC3B expression in vessels for autophagosome formation, we performed immunofluorescence staining of ovarian tissues from the OVX rats. The expression of CD31 and LC3B was localized in vessels of ovarian tissues (Fig. 3g). Quantification of LC3B-positive signals versus CD31-positive signals showed that the expression of LC3B in vessels was significantly decreased in the NTx groups compared to the normal group and substantially increased in the PRL-1 Tx groups compared to the NTx and Naïve Tx groups (Fig. 3h; *p < 0.05). These data indicated that transplanted PRL-1-overexpressing PD-MSCs induced the autophagic signaling pathway in the vessels of the ovaries of the OVX rats. Therefore, we confirmed the correlation between pericyte recruitment and autophagy (Fig. 3h). Interestingly, the immunofluorescence expression of NG2+ and LC3B+ showed higher co-expression with CD31+ in PRL-1 Tx groups compared to Naïve Tx groups. The constant value was 0.6040, confirming this correlation (not data shown). These data indicated that transplanted PRL-1-overexpressing PD-MSCs induced autophagy by pericyte recruitment in the vessels of the ovaries of the OVX rats.

### PRL-1-overexpressing PD-MSCs transplantation restored vascular remodeling by decreasing vascular permeability

**Fig. 4:**
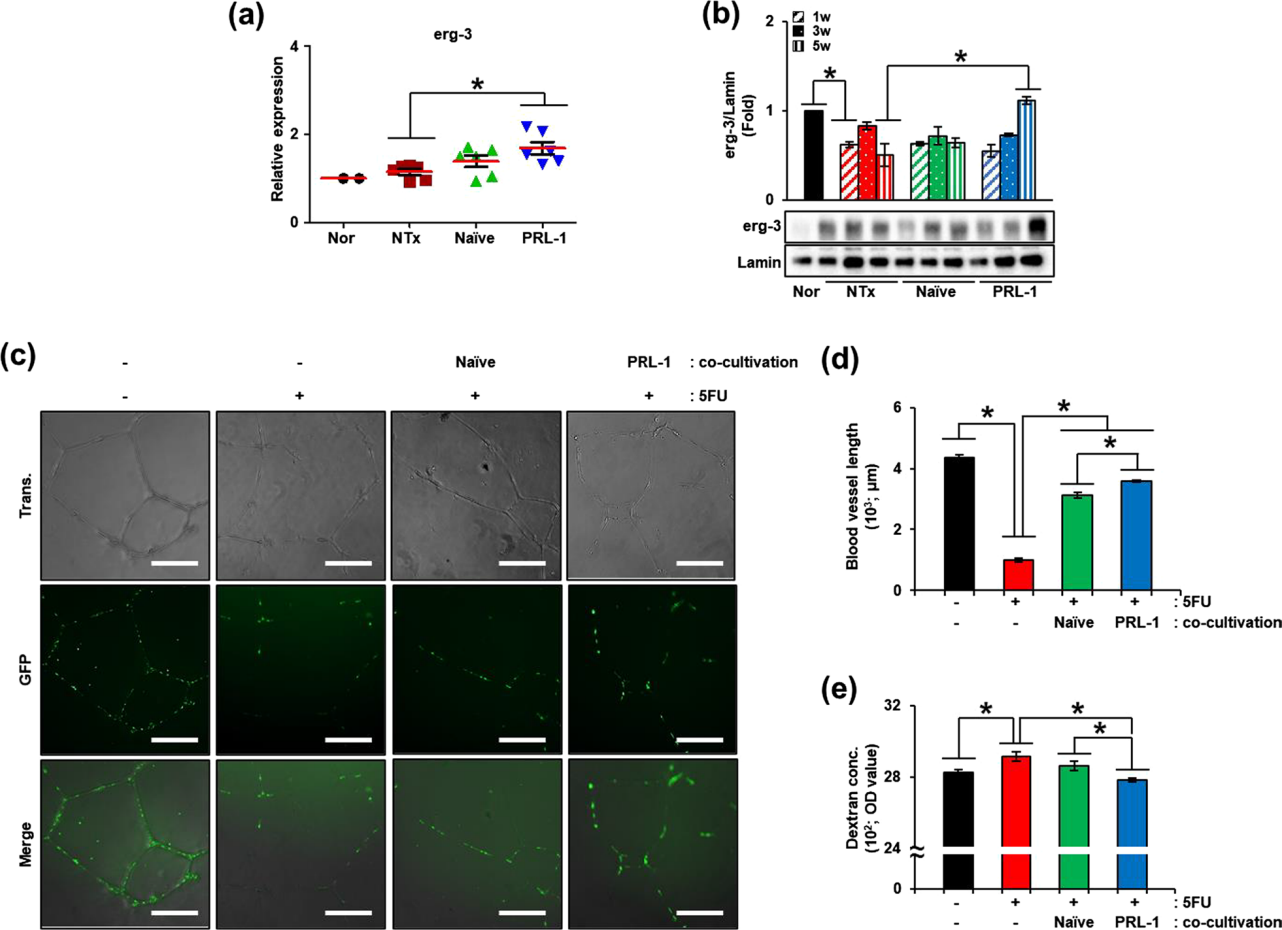
Effect of transplanted PRL-1-overexpressing cells on vascular permeability in the ovaries of OVX rats. **a** The mRNA expression of erg-3 in ovaries was analyzed by qRT-PCR. **b** The gene expression of erg-3 in the ovary was analyzed by western blots. **c** The vascular formation of HUVECs by PRL-1 was analyzed by DiI staining. **d** The vascular length of HUVECs was analyzed by ImageJ. **e** The vascular permeability of HUVECs altered by PRL-1 was analyzed by dextran assay. The data are representative of three independent experiments and expressed as the mean ± S.D. *p < 0.05, normal versus NTx, NTx versus Tx (Naïve and PRL-1), and Naïve versus PRL-1 at each time point

To analyze the vascular function improvement mediated by vascular regeneration, we analyzed vascular permeability factors [e.g., early growth response 3 (erg-3)] in the ovaries of the OVX rats. The mRNA expression of erg-3, which is a transcription factor that regulates vascular permeability and vascular homeostasis, was increased in the PRL Tx groups compared to the NTx groups and the Naïve Tx groups (Fig. 4a; *p < 0.05). Additionally, the protein expression of erg-3 in isolated nuclei of ovaries was increased in the PRL Tx groups compared to the NTx groups and the Naïve Tx groups (Fig. 4b; *p < 0.05).

To demonstrate the effect of PRL-1 by focusing on endothelial cells, we performed in vitro experiments using HUVECs. After 5-fluorouracil (5-FU) treatment of HUVECs, Naïve and PRL-1-overexpressing cells were cocultured with HUVECs using insert systems for 48 h. As shown in Fig. 4c, the tube formation of the endothelial cells showed substantial disruption of bridges and branching points without coculture with HUVECs and strong organization of bridges and branching points in Naïve and PRL-1-overexpressing cells cocultured with HUVECs (Fig. 4c, d; *p < 0.05). Moreover, to confirm the restoration of vascular function, we analyzed vascular permeability using a dextran assay at 48 h. Vascular permeability was increased in the non-cocultivation group compared to the normal group and decreased in the Naïve and PRL-1 overexpressing cellscocultivation group compared to the non-cocultivation group. Also, it was significantly decreased compared to Naïve cocultivation group in PRL-1 overexpressing cellscocultivation groups (Fig. 4e; *p < 0.05). These data indicated that transplanted PRL-1-overexpressing PD-MSCs enhanced vascular function through an activated regulator of vascular permeability in the OVX rats.

### PRL-1-overexpressing PD-MSCs transplantation enhanced ovarian function through sex hormone levels and follicular development in an OVX rat model

**Fig. 5:**
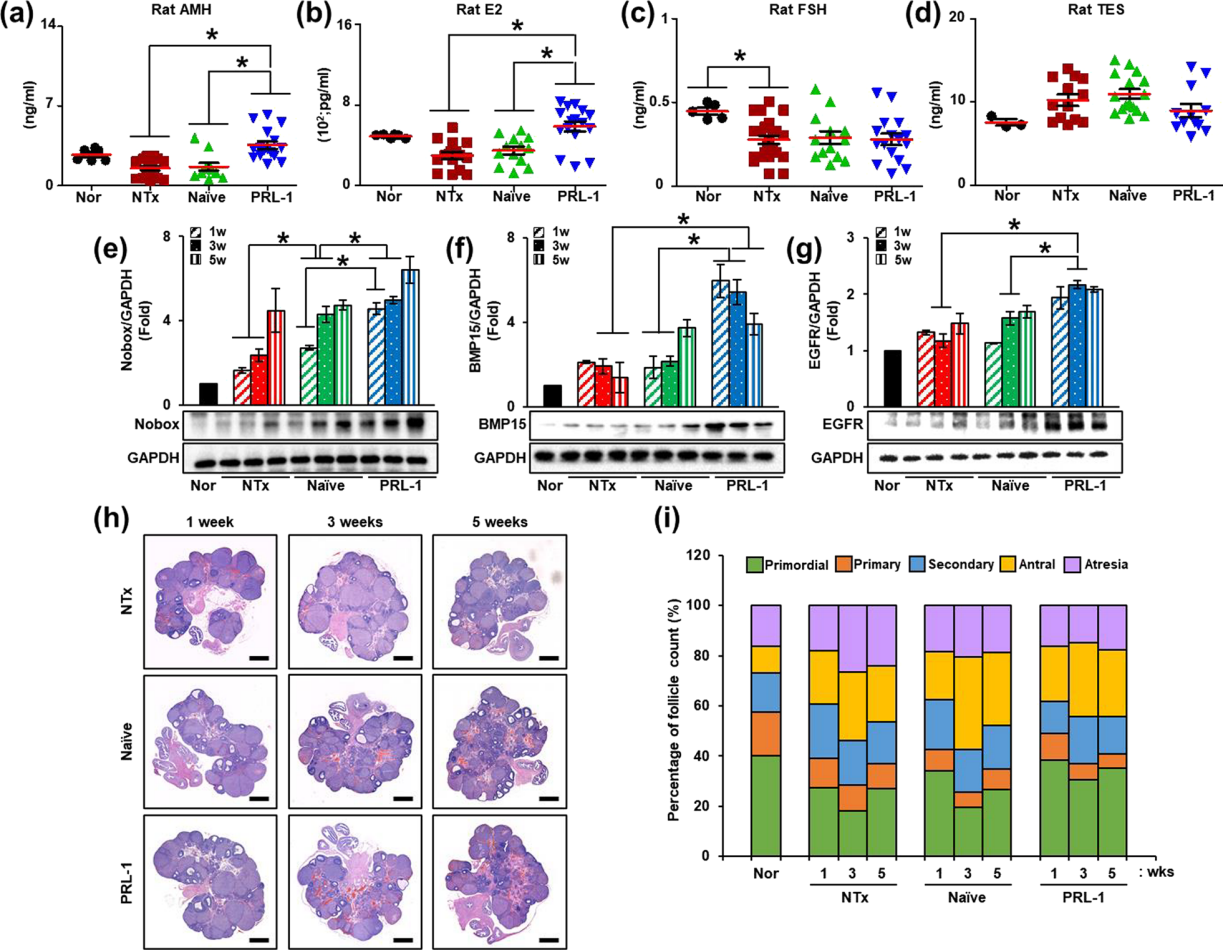
Effect of transplanted PRL-1-overexpressing cells on ovarian function in OVX rats. **a** The levels of AMH, **b** estrogen, **c** FSH and **d** testosterone in individual serum samples were analyzed by ELISAs. **e** The gene expression of Nobox, **f** BMP15 and **g** EGFR in the ovary was analyzed by western blots. **h** Histological analysis of follicular development was analyzed by H&E staining. Scale bar: 1 mm, magnification: 1.4×. **i** The percentage of follicles according to follicular development was analyzed by the 3D HISTECH program. The data are representative of three independent experiments and expressed as the mean ± S.D. *p < 0.05, normal versus NTx, NTx versus Tx (Naïve and PRL-1), and Naïve versus PRL-1 at each time point

In addition to regulating ovarian functions, the balance of endocrinal hormones is required for follicular development. To confirm the effect of PRL-1 on endocrinal hormones, we analyzed the levels of reproductive hormones, such as anti-Mullerian hormone (AMH), estradiol (E2), FSH and testosterone (TES), in the serum of the OVX rats using enzyme-linked immunosorbent assays (ELISAs). As a result, the levels of AMH and E2, which are involved in ovarian reserve and function, in individual serum samples were decreased in the NTx group compared to the normal group and significantly increased in the PRL-1 group compared to the NTx and Naïve Tx groups (Fig. 5a, b; *p < 0.05). However, the level of FSH in individual serum samples was decreased in the NTx, Naïve and PRL-1 Tx groups compared to the normal group and showed no differences among the groups (Fig. 5c; *p < 0.05). In contrast, the level of TES in individual serum samples was increased in the NTx and Naïve Tx groups compared to the normal group and decreased in the PRL-1 Tx groups compared to the NTx groups (Fig. 5d). Additionally, we analyzed the factors related to follicular development in the ovaries of the OVX rats. As a result, the protein expression of the newborn ovary homeobox gene (Nobox), which is an oocyte-specific gene involved in early follicular development, was found to be increased in the ovaries of the OVX rats. In particular, the protein expression of Nobox was significantly increased in the PRL-1 Tx groups compared to the Naïve Tx groups (Fig. 5e; *p < 0.05). Additionally, the protein expression of bone morphogenetic protein 15 (BMP15) and epidermal growth factor receptor (EGFR), which is involved in follicle maturation in late follicular development, was increased in the NTx groups compared to the normal group. In particular, the protein expression of BMP15 and EGFR was significantly increased in the PRL-1 Tx groups compared to the NTx and Naïve Tx groups (Fig. 5f, g; *p < 0.05).

To confirm the effect of PRL-1 on follicular development, we stained serially sectioned ovarian tissues from each group using H&E and analyzed the samples using the 3D HISTECH program. Matured follicles were different in each group (Fig. 5h). To quantify the ovarian follicles according to maturation stage, we counted the follicles in the stained tissues. In the NTx groups, decreased primordial follicles and increased follicular atresia were observed compared to those in the normal groups. In the Naïve and PRL-1 Tx groups, primordial follicles were recovered compared to those in the NTx group. Interestingly, follicular atresia was decreased in the PRL-1 Tx groups compared to the NTx and Naïve Tx groups. Unexpectedly, follicles in the other stages (e.g., primary, secondary and antral follicles) were not significantly different among the groups (Fig. 5i, Additional file 1: Table S2). These results indicated that transplanted PRL-1-overexpressing cells improved follicular development and hormone levels in the OVX rats.

### Effect of vascular remodeling and follicular development of the PRL-1 overexpressing PD-MSCs treated with siPRL-1 or PDGF inhibitor and recombinant PDGF-BB ex vivo

**Fig. 6:**
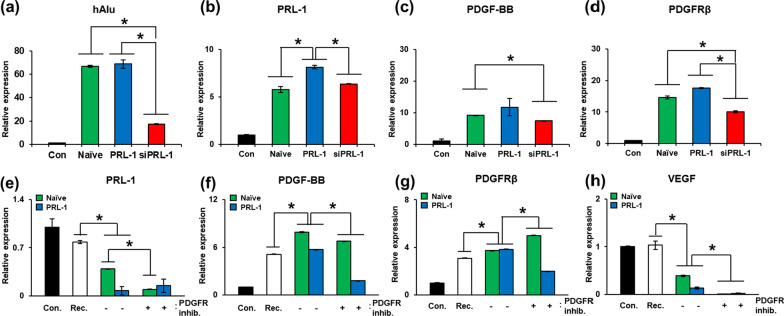
Effect of PRL-1-overexpressign cells on follicular development via the PDGF pathway ex vivo. **a** The mRNA expression of hAlu, **b** human PRL-1, **c** PDGF-BB, **d** PDGFRβ, **e** Nobox and **f** Lhx8 in ovaries with siRNA-PRL-1 was analyzed by qRT-PCR. **g** The mRNA expression of PDGF-BB, **h** PDGFRβ, **i** VEGF and VEGFR2 in ovaries treated with recombinant PDGF and inhibitor was analyzed by qRT-PCR. The data are representative of three independent experiments and expressed as the mean ± S.D. *p < 0.05, normal versus NTx, NTx versus Tx (Naïve and PRL-1), Naïve versus PRL-1 at each time point

To analyze the effect of PRL-1-overexpressing PD-MSCs transplantation in the ovaries of the OVX rats, we performed an ex vivo experiment using siRNA-PRL-1. We analyzed the mRNA expression of the human-specific Alu (hAlu) sequence in the injected ovaries to confirm the engraftment activity of human PD-MSCs. The mRNA expression of the hAlu sequence was strongly increased in the Naïve and PRL-1 injection groups. However, the mRNA expression of the hAlu sequence was significantly decreased in the siRNA-PRL-1 injection groups compared to the Naïve and PRL-1 injection groups (Fig. 6a; *p < 0.05). We analyzed the mRNA expression of human PRL-1 in ovarian tissues to confirm the expression of injected PRL-1. Interestingly, the mRNA expression of human PRL-1 was at the basal level in the Naïve injection groups. The mRNA expression of human PRL-1 was substantially increased in the PRL-1 injection groups compared to the control and Naïve injection groups. However, the mRNA expression of human PRL-1 was significantly decreased in the siRNA-PRL-1 injection groups compared to the PRL-1 injection groups (Fig. 6b; *p < 0.05). Additionally, we analyzed the mRNA expression of the PDGF family in ovarian tissues. The mRNA expression of PDGF-BB, which is a classic PDGF ligand related to vascular remodeling, was significantly increased in the Naïve and PRL-1 injection groups compared to the control groups. The mRNA expression of PDGF-BB was decreased in the siRNA-PRL-1 group compared to the Naïve and PRL-1 injection groups (Fig. 6c; *p < 0.05). The mRNA expression of PDGFRβ, which is a representative PDGF receptor related to vascular remodeling, was strongly increased in the Naïve and PRL-1 injection groups compared to the control groups. The mRNA expression of PDGFRβ was significantly decreased in the siRNA-PRL-1 injection groups compared to the Naïve and PRL-1 injection groups (Fig. 6d; *p < 0.05).

To analyze the effect of PDGF via transplantation of PRL-1-overexpressing PD-MSCs in the ovaries of the OVX rats, we performed an ex vivo experiment using imatinib as a PDGFR inhibitor and recombinant PDGF-BB. We analyzed the mRNA expression of human PRL-1 in ovarian tissues to confirm the expression of cocultivated PRL-1-overexpressing cells. The groups with PRL-1-overexpressing PD-MSCs and PDGFR inhibitor showed increased mRNA expression of human PRL-1 compared to the PRL-1 groups (Fig. 6e; *p < 0.05). These data indicated that PRL-1 affects PDGF signaling, whereas PDGF signaling does not affect PRL-1. We analyzed the mRNA expression of the PDGF family in cocultivated ovaries. The mRNA expression of PDGF-BB was significantly increased in the Naïve and PRL-1 cocultivation groups compared to the control and recombinant PDGF-BB + Tx groups. The cocultivated groups with Naïve and PRL-1-overexpressing PD-MSCs treated with PDGF receptor inhibitor showed significantly decreased mRNA expression of PDGF-BB compared to the Naïve and PRL-1 cocultivation groups (Fig. 6f; *p < 0.05). The mRNA expression of PDGFRβ was significantly increased in the Naïve and PRL-1 cocultivation groups compared to the control and recombinant PDGF-BB Tx groups. Compared to PRL-1-overexpressing PD-MSC cocultivation, cocultivation with PRL-1-overexpressing PD-MSCs treated with PDGF receptor inhibitor significantly decreased the mRNA expression of PDGFRβ (Fig. 6g; *p < 0.05). In addition, we analyzed VEGF signaling in vascular remodeling in cocultivated di-sectioned ovaries. The mRNA expression of VEGF was decreased in the Naïve and PRL-1 groups compared to the recombinant PDGF-BB cocultivation groups. The mRNA expression of VEGF was significantly decreased in the Naïve and PRL-1 cocultivation groups compared to the Naïve and PRL-1 cocultivation groups (Fig. 6h; *p < 0.05). The mRNA expression of VEGFR2 showed no difference among the groups excluding the control group (data not shown). These results indicate that VEGF binds PDGF receptors, but VEGFR2 is a different signaling pathway from the PDGF signaling pathway [22].

## Discussion

Vascular remodeling is essential and important for the regeneration of damaged organs as well as organogenesis. This process plays a role in delivering oxygen as well as various nutrients that (1) enlarge the vascular network in ischemic tissues for therapeutic purposes by angiogenesis and (2) invade the vascular network to enable progenitor survival and differentiation [23]. Although vascular structure and blood flow are estrous cycle dependent in reproductive organs including uterus, but their changes in ovarian tissues are limited [24]. For the treatment of degenerative diseases, stem cells have been researched for their therapeutic mechanisms, and their paracrine effects were reported to be mediated by the secretion of proangiogenic factors such as PDGF, VEGF and FGFs [25, 26]. Moreover, MSC-based treatment of damaged tissues could enhance microvascular density with increasing tissue perfusion. Hence, various paracrine effects of MSCs can enhance vascular remodeling in infarction [27]. In particular, abnormalities in ovarian angiogenesis were observed in patients with PCOS. PCOS is a type of ovarian dysfunction with pathological characteristics, and clinical trials of various drugs for treatment of PCOS have been performed [28, 29].

Recently, Cho et al. and colleagues demonstrated that transplanted PD-MSCs improve ovarian function via the VEGF pathway in OVX rats [11]. However, there is a lack of research on the regenerative mechanism involved in vascular regeneration, and MSCs have a weaker mode of action (MOA) in improving ovarian function through vascular remodeling. To overcome these issues, many researchers have studied functionally enhanced stem cells for degenerative diseases [30, 31]. However, the therapeutic mechanism of functionally enhanced stem cells in rats with ovarian dysfunction has never been reported until now. In our study, we used functionally enhanced PD-MSCs for the treatment of ovarian dysfunction and to maximize their therapeutic efficacies in ovarian dysfunction model. Finally, we estimated the therapeutic mechanisms of PRL-1 on rats with ovarian dysfunction by stimulating vascular regeneration.

PRLs, including PRL-1, PRL-2 and PRL-3, are involved in cell migration together with differentiation for liver regeneration. Recently, these molecules were shown to be involved in the regulation of endothelial cell differentiation in the digestive system [32, 33]. Additionally, Kim et al. reported that PRL-1-enhanced PD-MSCs induced liver regeneration by vascular remodeling through alterative expressions of various miRNAs and targeted genes. The researchers found a slight correlation in which the expression of PDGFRα and PDGFRβ was increased in the PRL-1-enhanced PD-MSCs Tx groups compared to the PD-MSCs Tx groups [34]. Otherwise, researchers reported that PRL-1 inhibited endothelial cell differentiation by reducing the expression of adhesion molecules in inflammation-induced endothelial cells in cancer although their correlation between PRL-1 and PDGF still unclear on vascular remodeling [35]. Therefore, our data suggested that their correlation between PRL-1 and PDGF could be used as a fundamental evidence of vascular remodeling processing in order to restore injured tissues including ovary. Recent reports found higher PRL-1, PRL-2 and PRL-3 transcript levels in the vasculature and endothelial cells. Blockade of PRL-2 inhibited vascular remodeling by suppressing endothelial cell migration via the VEGF-A/NOTCH-1 signaling pathway in the postnatal mouse retina [15]. Also, Xu et al. demonstrated that PRL-3 correlated with VEGF in HUVECs downstream of VEGF/MEF2C [14]. Based on these reports, we focused not only on whether the paracrine effect of PRL-1 regulates vascular function and ovarian function but also on which factor mediated by PRL-1 is a major regulator in the OVX rats.

During vascular development, pericytes collaborate with endothelial cells and are major regulators of vascular remodeling, including stabilization and maturation. Specifically, these cells modulate the metastasis of endothelial cells (e.g., cell growth, proliferation, differentiation and migration) and smooth muscle cell contraction for capillary blood flow for vascular remodeling [36]. Our findings indicated that transplanted PD-MSCs overexpressing PRL-1 induced the expression of NG2, which is related to pericyte recruitment, and erg-3, which is related to vascular permeability, in the arteries of ovarian tissues. Additionally, several researchers have reported that PRL-1 is correlated with reproductive systems. PRL-1 is localized in the surface epithelium and oviduct of the ovary. Specifically, higher expression of PRL-1 was shown in the stroma, granulosa cells, corpus luteum, and vasculature with the medulla [37]. Several studies have reported that PRL-1 is regulated by FSH, and we also confirmed these results by demonstrating that transplanted PRL-1-overexpressing cells improved ovarian function in the OVX rats [13, 38].

In young adult age, ovariectomy affects alters follicle development but maintains ovarian function because increases atresia follicle but also increases compensatory antral follicle [39]. As a result, transplanted PRL-1-overexpressing PD-MSCs enhanced the concentration of AMH and estrogen as well as the expression of genes related to follicular development in the OVX rats. The follicles were counted, and PRL-1 resulted in increased primordial follicles and decreased follicular atresia in the OVX rats. Transplanted PRL-1-overexpressing PD-MSCs improved ovarian function through vascular remodeling compared to NTx group. However, the further study on the paracrine effect of PRL-1-enhanced PD-MSCs on vascular remodeling and ovarian dysfunction should be needed.

Basically, PDGF, as a proangiogenic factor, plays a role in vascular remodeling and steroid production for ovarian function, and it leads to mature and stable endothelial cells [20, 40]. So, we confirmed that PDGF-BB increased by PRL-1-enhanced PD-MSCs bind with their receptors and mediate the PDGF signaling pathway in cytokine array analysis and the expression of PDGFRβ in arteries and follicles of ovarian tissues. As shown in Fig. 2d, the expression and localization of PDGF were confirmed in arteries specific to the permeable theca layer of follicles in ovarian tissues and were increased in the PRL-1 group compared to the Naïve group. Despite these results, PDGF secreted by PRL-1-enhanced PD-MSCs showed higher expression in the arteries and theca layers of follicles. Based on previous results, we analyzed the correlation between PRL-1 and PDGF in the ovary to confirm the paracrine effect of PRL-1. In our ex vivo data, the lower level of PRL-1 after siRNA treatment inhibited the expression of PDGFRβ in the ovary. The expression of factors related to follicular development in ovarian tissues was decreased, and PRL-1 expression was suppressed in PD-MSCs by siRNA. In contrast, PDGF in PD-MSCs treated with recombinant PDGF or an inhibitor regulated the expression of PDGF-BB and PDGFRβ and the VEGF pathway in ovarian tissues. Therefore, our data suggested the paracrine effect of PRL-1 could be restored ovarian function in the OVX rats through activation of PDGF signaling pathway by their correlation between PRL-1 and PDGF.

In conclusion, our findings indicated that PRL-1-enhanced PD-MSCs regulated the vascular formation of HUVECs and secreted higher levels of PDGF than PD-MSCs in vitro. After PRL-1-enhanced PD-MSC transplantation into the OVX rats, vascular remodeling and pericyte recruitment were enhanced, along with the autophagic pathway. Moreover, transplanted PRL-1-enhanced PD-MSCs restored ovarian function, including reproductive hormone levels and follicular development, in the OVX rats. Therefore, these findings offer new insights into functionally enhanced stem cell therapy for reproductive medicines.

## Conclusion

**Fig. 7:**
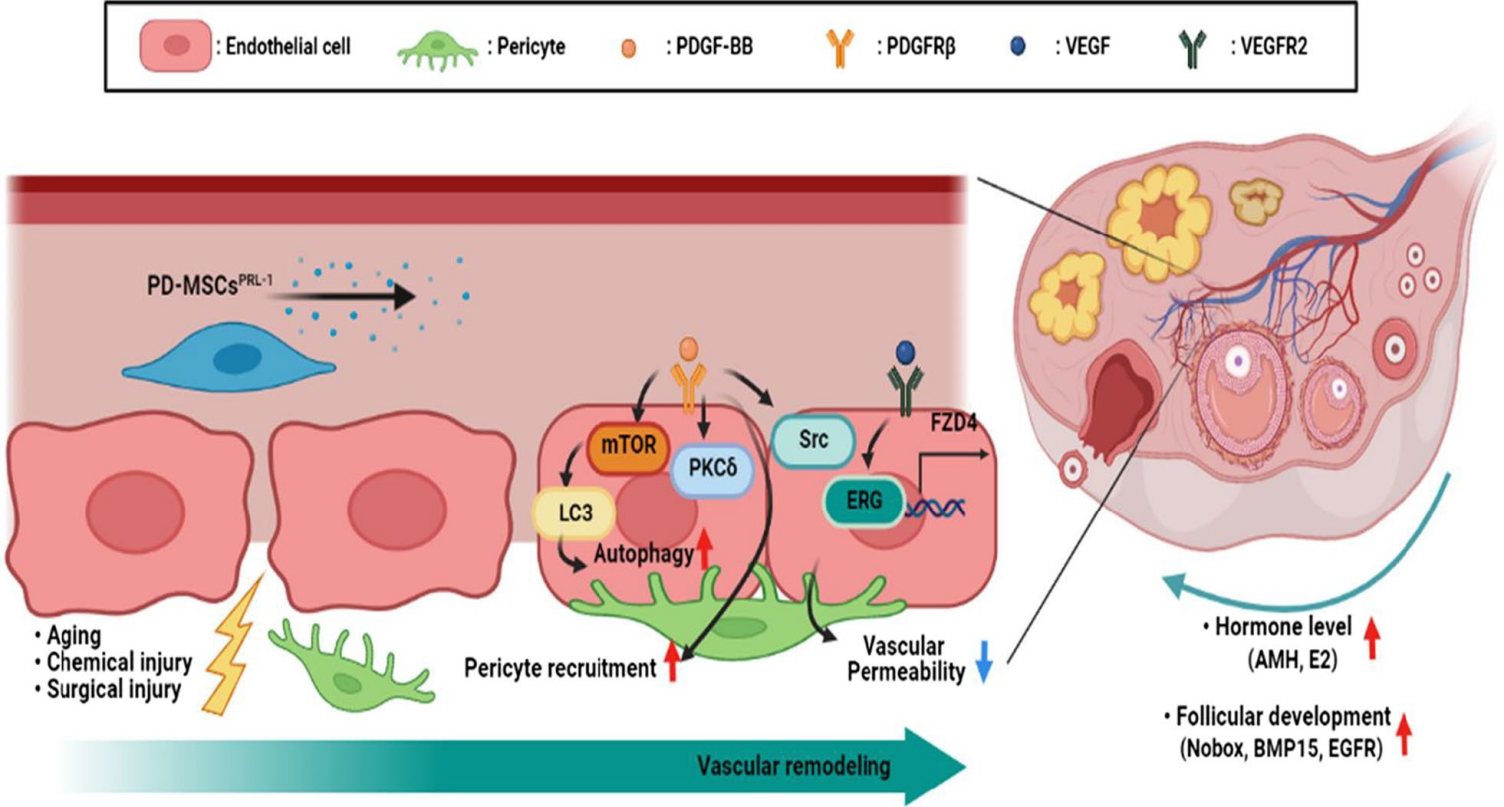
Graphical abstract describes that PRL-1 activates PDGF signaling to improve vascular remodeling in ovarian, thereby improving ovarian function. *PD-MSCs* placenta-derived mesenchymal stem cells, *PRL-1* phosphatase of regenerating liver-1, *PDGF-BB* platelet-derived growth factor-BB, *PDGFRβ* platelet-derived growth factor receptor beta, *VEGF* vascular endothelial growth factor, *VEGFR2* vascular endothelial growth factor receptor 2, *mTOR* mammalian target of rapamycin, *LC3* microtube-associated protein 1 light chain 3, *PKCδ* protein kinase C-delta, *Src* proto-oncogene tyrosine-protein kinase Src, *ERG* ETS-related gene, *FZD4* frizzled class receptor 4, *AMH* anti-Mullerian hormone, *E2* estrogen, *Nobox* newborn ovary homeobox, *BMP15* bone morphogenetic protein 15, *EGFR* epidermal growth factor receptor

In conclusion, PRL-1 restore ovarian function by enhanced vascular function through PDGF signaling pathway (Fig. 7). These findings offer new insight into the effects of functionally enhanced stem cell therapy for reproductive systems and should provide new avenues to develop more efficient therapies in degenerative medicine.

## Supplementary Information

**Additional file 1.** **Supplementary Table 1.** Primer sequences using quantitative real time polymerase chain reaction. **Supplementary Table 2.** Comparison of follicle counts after transplantation in vivo. **Supplementary Fig. 1.** The level of PDGF in PRL-1 compared to Naïve. (a-b) The intensity of PDGF-BB was expressed dot blot by cytokine array and Image J program. The data were representative of three independent experiments and expressed as means S.D. Significant indicates * *p* < 0.05, Naïve vs. PRL-1. **Supplementary Fig. 2.** Effect of PRL-1 on PDGF downstream in ovary of OVX rats. (a) The mRNA expression of PKCδ in ovary was analyzed by qRT-PCR. (b) The gene expression and localization of p-Src in ovary were analyzed by IF staining. (c) The mRNA expression of VEGF and (d) VEGFR2 in ovary were analyzed by qRT-PCR. (e) The gene expression of VEGFR in ovary was analyzed by western blot. The data were representative of three independent experiments and expressed as means S.D. Significant indicates * *p* < 0.05, Normal vs. NTx, NTx vs. Tx (Naive and PRL-1), Naive vs. PRL-1 in each time point. **Supplementary Fig. 3.** Effect of PRL-1 on angiogenesis in ovary of OVX rats. (a) The mRNA expression of HIF1α and (b) Endoglin in ovary were analyzed by qRT-PCR. (c) The gene expression of HIF1α and (d) Endoglin in ovary were analyzed by western blot. The data were representative of three independent experiments and expressed as means S.D. Significant indicates * *p* < 0.05, Normal vs. NTx, NTx vs. Tx (Naive and PRL-1), Naive vs. PRL-1 in each time point.

## Data Availability

The data that support the findings of this study are available from the corresponding author upon reasonable request.

## Abbreviations

POF: Premature ovarian failure
PCOS: Polycystic ovary syndrome
HRT: Hormone replacement therapy
MOA: Mode of action
OVX: Ovariectomy
PD-MSCs: Placenta-derived mesenchymal stem cells
PRL-1: Phosphatase of regenerating liver-1, PD-MSCs^PRL^^-^^1^
hAlu: Human specific Alu
PDGF: Platelet-derived growth factor
VEGF: Vascular endothelial growth factor
FGF: Fibroblast growth factor
H&E staining: Hematoxylin and eosin staining
ELISAs: Enzyme-linked immunosorbent assays
NTx: Non-transplantation
PKCδ: Protein kinase C-delta
Src: Proto-oncogene tyrosine-protein kinase Src
CD31: Platelet endothelial cell adhesion molecule (PECAM-1)
NG2: Neuron-glial antigen 2
mTOR: Mammalian target of rapamycin
LC3B: Microtube-associated protein 1 light chain 3
GAPDH: Glyceraldehyde 3-phosphate dehydrogenase
HUVECs: Human umbilical vein endothelial cells
5FU: 5-Fluorouracil
AMH: Anti-Mullerian hormone
E2: Estrogen
FSH: Follicle stimulating hormone
TES: Testosterone
Nobox: Newborn ovary homeobox
BMP15: Bone morphogenetic protein 15
EGFR: Epidermal growth factor receptor
