# Supplementary material for: Increased phosphatase regenerating liver-1 trigger vascular remodeling in injured ovary via platelet-derived growth factor signaling pathway
Source: Stem Cell Res Ther. 2022 Mar 7;13:95. doi: 10.1186/s13287-022-02772-9 (PMC8900363; doi:10.1186/s13287-022-02772-9)
Supplement: Supplementary file 1 — Additional file 1. Supplementary Table 1. Primer sequences using quantitative real time polymerase chain reaction. Supplementary Table 2. Comparison of follicle counts after transplantation in vivo. Supplementary Fig. 1. The level of PDGF in PRL-1 compared to Naïve. (a-b) The intensity of PDGF-BB was expressed dot blot by cytokine array and Image J program. The data were representative of three independent experiments and expressed as means S.D. Significant indicates * p < 0.05, Naïve vs. PRL-1. Supplementary Fig. 2. Effect of PRL-1 on PDGF downstream in ovary of OVX rats. (a) The mRNA expression of PKCδ in ovary was analyzed by qRT-PCR. (b) The gene expression and localization of p-Src in ovary were analyzed by IF staining. (c) The mRNA expression of VEGF and (d) VEGFR2 in ovary were analyzed by qRT-PCR. (e) The gene expression of VEGFR in ovary was analyzed by western blot. The data were representative of three independent experiments and expressed as means S.D. Significant indicates * p < 0.05, Normal vs. NTx, NTx vs. Tx (Naive and PRL-1), Naive vs. PRL-1 in each time point. Supplementary Fig. 3. Effect of PRL-1 on angiogenesis in ovary of OVX rats. (a) The mRNA expression of HIF1α and (b) Endoglin in ovary were analyzed by qRT-PCR. (c) The gene expression of HIF1α and (d) Endoglin in ovary were analyzed by western blot. The data were representative of three independent experiments and expressed as means S.D. Significant indicates * p < 0.05, Normal vs. NTx, NTx vs. Tx (Naive and PRL-1), Naive vs. PRL-1 in each time point. [file 13287_2022_2772_MOESM1_ESM.docx]

**Supplementary Table 1 Primer sequences using quantitative real time polymerase chain reaction.**

| **Gene** | **Primer** | **Annealing  Temperature**  **(℃)** | | **NM number** |
| --- | --- | --- | --- | --- |
| hAlu | F: 5’-GGA GGC TGA GGC AGG AGA A-3’ | 60 | NM_002715 | |
|  | R: 5’-CGG AGT CTC GCT CTG TCG CCC A-3’ |  |  |  |
| hPRL-1 | F: 5’-TAC TGC TCC ACC AAG AAG CC-3’ | 64.3 | NM_001385254.1 | |
|  | R: 5’-AGG TTT ACC CCA TCC AGG TC-3’ |  |  |  |
| PDGFBB | F: 5'-CTC AGA GAG ATG GAG GTG CTC TC-3' | 60 | NM_031524.1 | |
|  | R: 5'-GCC CAG AGG AGT TCA TGT CTT AT-3' |  |  |  |
| PDGFRα | F: 5'-GAG GAC GAT TCT GCC ATC AT-3' | 58 | NM_012802.1 | |
|  | R: 5'-CAG TTC TGA CGT GGC TTT CA-3' |  |  |  |
| PDGFRβ | F: 5'-TGT TCG TGC TAT TGC TCC TG-3' | 58 | NM_031524.1 | |
|  | R: 5'-TGT CAG CAC ACT GGA GAA GG-3' |  |  |  |
| VEGF | F: 5'-ACT GGA CCC TGG CTT TAC TG-3' | 59 | NM_001110333.2 | |
|  | R: 5'-ACG CAC TCC AGG GCT TCA TC-3' |  |  |  |
| VEGFR2 | F: 5'-AAG CAA ATG CTC AGC AGG AT-3' | 58 | NM_013062.2 | |
|  | R: 5'-TAG GCA GGG AGA GTC CAG AA-3' |  |  |  |
| HIF1α | F: 5'-TCG GCG AAG TAA AGA ATC TGA A-3' | 56 | NM_024359.1 | |
|  | R: 5'-CAA ATC ACC AGC ATC CAG AAG-3' |  |  |  |
| Endoglin | F: 5'-AAG GTG TGA CTG TAC ACA AG-3' | 56 | NM_001010968.3 | |
|  | R: 5'-CCA GAT CTG CAT ATT GTG GT-3' |  |  |  |
| PKCδ | F: 5'-AAG CAT TCA ACG CCA GGT TC-3' | 60 | NM_198780.3 | |
|  | R: 5'-GGG CGA GTC TGT CAG CTC AAT-3' |  |  |  |
| Erg-3 | F: 5’-CAT GCT AGA AAC ACA GAT TTA CCT T-3’ | 60 | NM_133397.2 | |
|  | R: 5’- ACC TGG ATT AGC AAG GCG AC-3’ |  |  |  |
| Nobox | F : 5’- AGC CAG TGC AGA TCT GCA CCG-3’ | 60 | NM_001192013.1 | |
|  | R : 5’- TGT CAC TGC CAG GAA CAT CCC TC-3’ |  |  |  |
| Lhx8 | F : 5’- GTA TCA CTT GGC TTG CTT-3’ | 56 | NM_001012219.2 | |
|  | R : 5’- ATT ACC GTT CTC CAC TTC-3’ |  |  |  |
| GAPDH | F: 5'-TCC CTC AAG ATT GTC AGC AA-3' | 55 | NM_017008.4 | |
|  | R: 5'-AGA TCC ACA ACG GAT ACA TT-3' |  |  |  |

The mRNA expression was analyzed by the sequences of Table S1.

**Supplementary Table 2 Comparison of follicle counts after transplantation *in vivo*.**

|  | | Primordial  (%) | Primary  (%) | Secondary  (%) | Antral  (%) | Atresia  (%) |
| --- | --- | --- | --- | --- | --- | --- |
| Normal | | 39.92±2.42 | 17.72±0.60 | 14.88±0.70 | 10.02±0.77 | 17.45±0.99 |
| NTx | 1w | 27.48±3.44 | 11.65±0.10* | 21.54±5.43 | 21.20±1.94* | 18.13±6.26 |
|  | 3w | 18.08±2.45* | 10.35±1.90* | 17.73±2.52 | 27.28±4.86* | 26.57±1.35* |
|  | 5w | 26.96±3.50* | 9.88±1.39* | 16.81±6.89 | 22.35±2.38* | 24.00±5.17 |
| Naïve | 1w | 33.92±2.60** | 8.83±2.30 | 19.74±2.22 | 19.02±1.68 | 18.49±3.46 |
|  | 3w | 16.70±5.63 | 6.04±0.53** | 17.53±3.68 | 38.56±9.11 | 21.17±2.78** |
|  | 5w | 26.20±7.61 | 8.04±1.28 | 17.20±3.19 | 29.84±7.26 | 18.72±4.35 |
| PRL-1 | 1w | 38.30±2.16** | 10.61±1.20 | 12.71±2.11** | 22.20±2.49 | 16.18±4.70 |
|  | 3w | 30.59±4.16**, # | 6.32±1.22** | 18.91±4.01 | 29.36±2.52 | 14.82±0.60**, # |
|  | 5w | 35.03±3.69** | 5.73±0.79** | 15.02±2.67 | 26.46±1.15 | 17.76±0.60** |

*, NTx vs. Nor (*p*<0.05). **, Tx vs. NTx (*p*<0.05). #, Naïve vs. PRL-1.

**
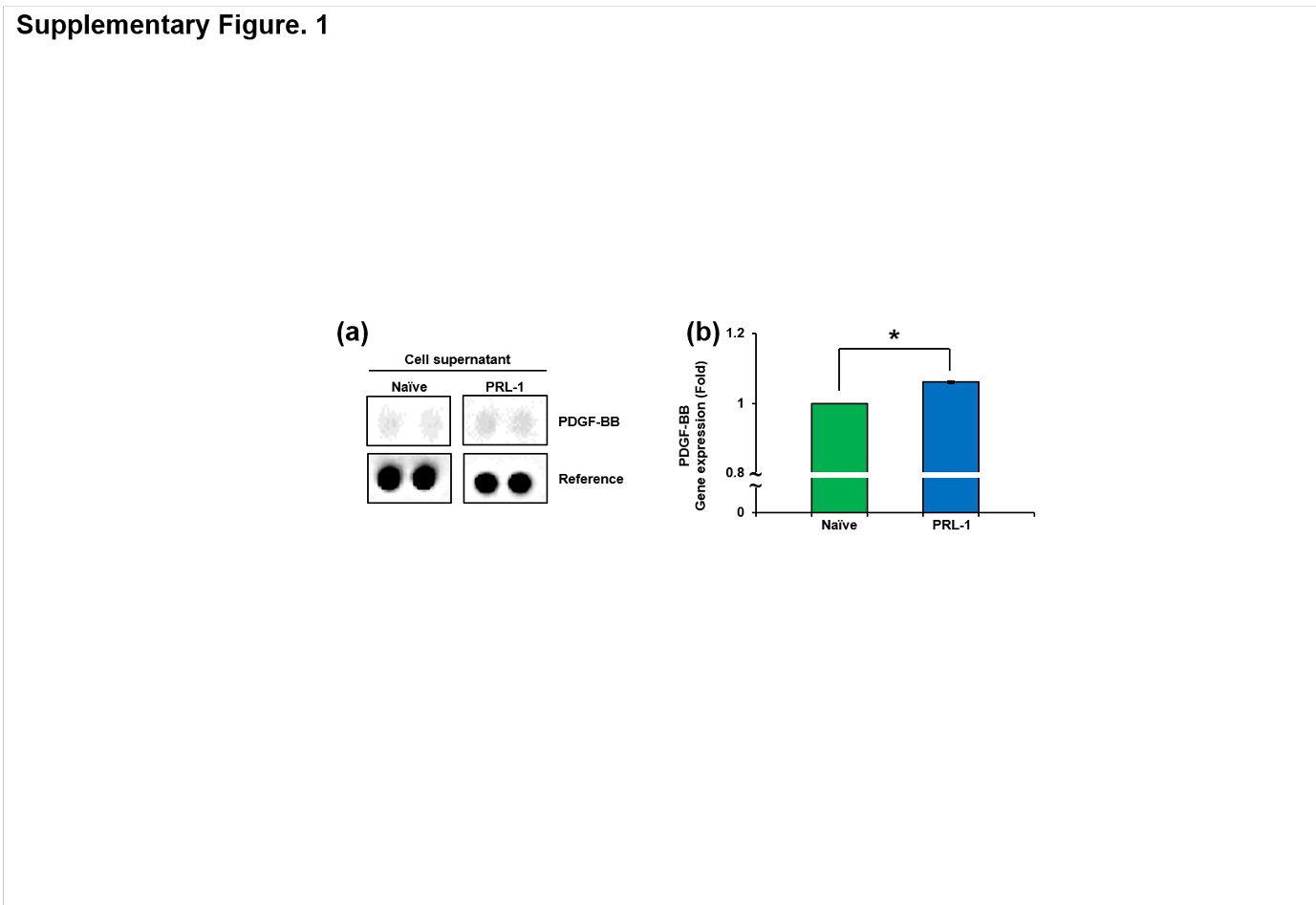
Supplementary Fig.1** The level of PDGF in PRL-1 compared to Naïve. (a-b) The intensity of PDGF-BB was expressed dot blot by cytokine array and Image J program. The data were representative of three independent experiments and expressed as means $\pm$ S.D. Significant indicates * *p*<0.05, Naïve vs. PRL-1

**
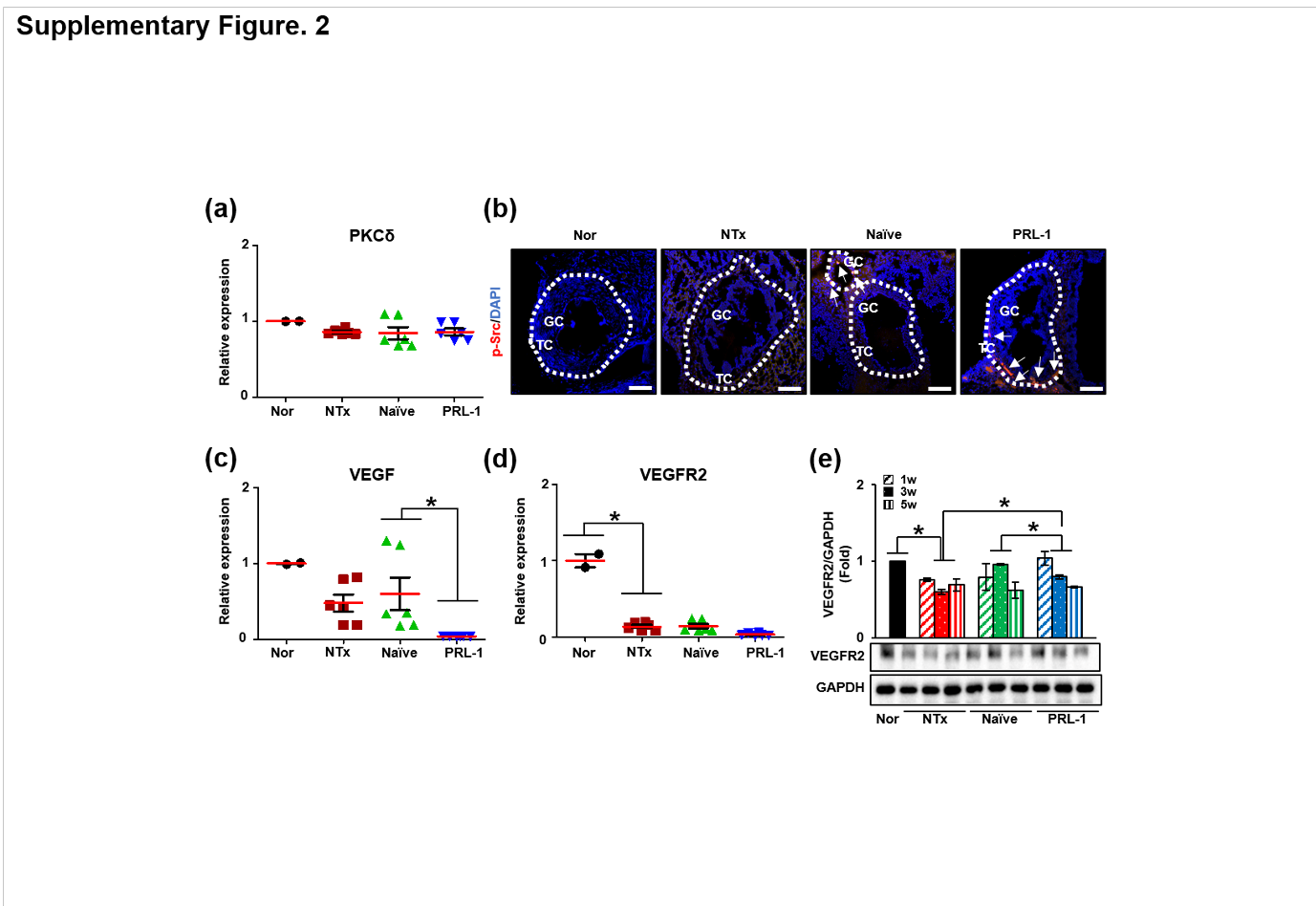
Supplementary Fig.2** Effect of PRL-1 on PDGF downstream in ovary of OVX rats. (a) The mRNA expression of PKCδ in ovary was analyzed by qRT-PCR. (b) The gene expression and localization of p-Src in ovary were analyzed by IF staining. (c) The mRNA expression of VEGF and (d) VEGFR2 in ovary were analyzed by qRT-PCR. (e) The gene expression of VEGFR in ovary was analyzed by western blot. The data were representative of three independent experiments and expressed as means $\pm$ S.D. Significant indicates * *p*<0.05, Normal vs. NTx, NTx vs. Tx (Naive and PRL-1), Naive vs. PRL-1 in each time point.

**
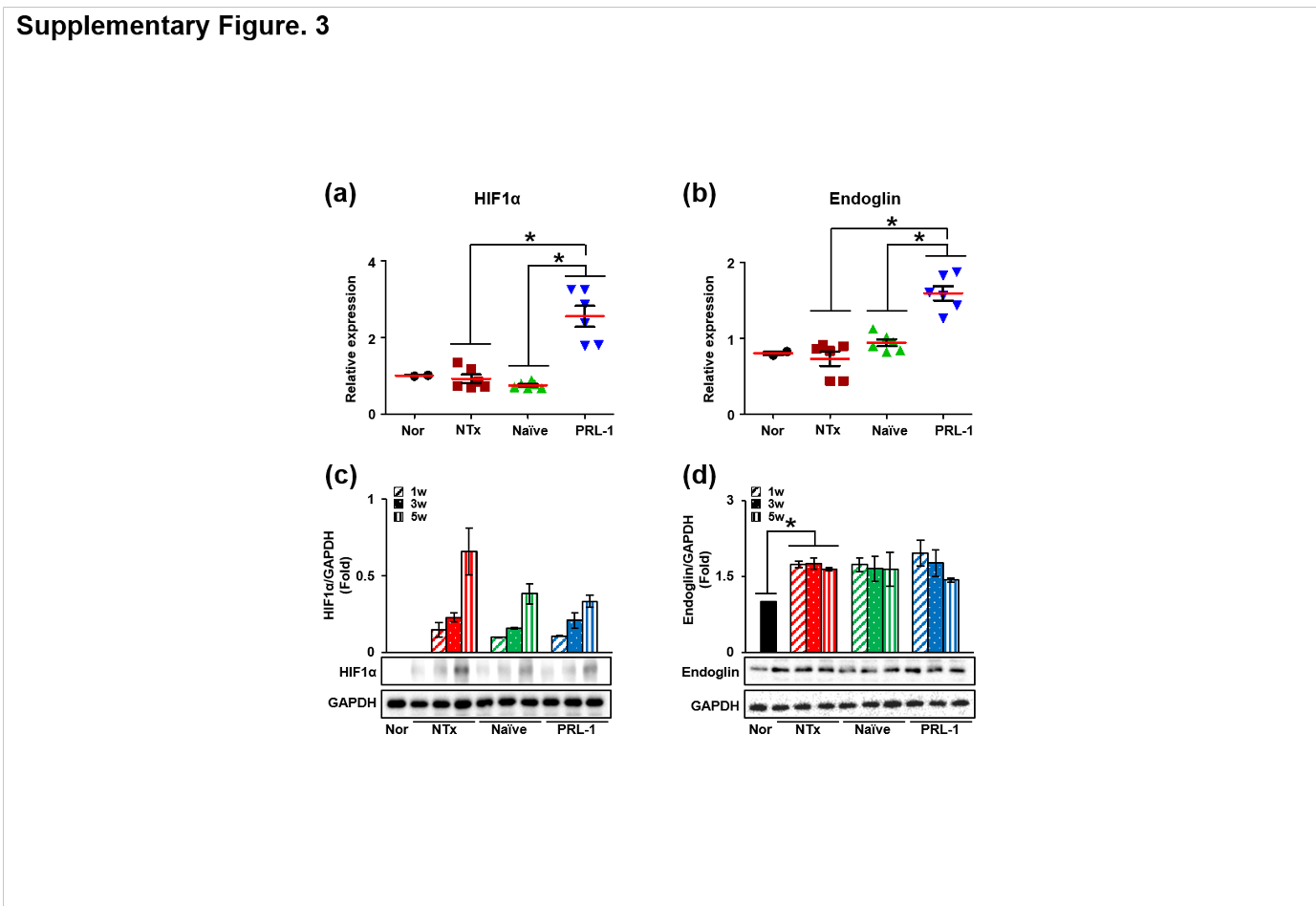
Supplementary Fig.3** Effect of PRL-1 on angiogenesis in ovary of OVX rats. (a) The mRNA expression of HIF1α and (b) Endoglin in ovary were analyzed by qRT-PCR. (c) The gene expression of HIF1α and (d) Endoglin in ovary were analyzed by western blot. The data were representative of three independent experiments and expressed as means $\pm$ S.D. Significant indicates * *p*<0.05, Normal vs. NTx, NTx vs. Tx (Naive and PRL-1), Naive vs. PRL-1 in each time point.
